# Inheritance and Establishment of Gut Microbiota in Chickens

**DOI:** 10.3389/fmicb.2017.01967

**Published:** 2017-10-10

**Authors:** Jinmei Ding, Ronghua Dai, Lingyu Yang, Chuan He, Ke Xu, Shuyun Liu, Wenjing Zhao, Lu Xiao, Lingxiao Luo, Yan Zhang, He Meng

**Affiliations:** ^1^Shanghai Key Laboratory of Veterinary Biotechnology, Department of Animal Science, School of Agriculture and Biology, Shanghai Jiao Tong University, Shanghai, China; ^2^Carilion Clinic, Roanoke, VA, United States

**Keywords:** gut microbiota, inheritance, establishment, 16S rRNA, chicken

## Abstract

In mammals, the microbiota can be transmitted from the placenta, uterus, and vagina of the mother to the infant. Unlike mammals, development of the avian embryo is a process isolated from the mother and thus in the avian embryo the gut microbial developmental process remains elusive. To explore the establishment and inheritance of the gut microbiome in the avian embryo, we used the chicken as the model organism to investigate the gut microbial composition in embryos, chicks, and maternal hens. We observed: (1) 28 phyla and 162 genera of microbes in embryos where the dominated genus was *Halomonas* (79%). (2) 65 genera were core microbiota in all stages with 42% and 62% gut microbial genera of embryo were found in maternal hen and chick, respectively. There was a moderate correlation (0.40) between the embryo and maternal, and 0.52 between the embryo and chick at the family level. (3) Gut microbes that are involved in substance metabolism, infectious disease, and environmental adaptation are enriched in embryos, chicks, and maternal hens, respectively. (4) 94% genera of gut microbial composition were similar among three different chicken breeds which were maintained under similar conditions. Our findings provide evidence to support the hypothesis that part of the microbial colonizers harbored in early embryos were inherited from maternal hens, and the gut microbial abundance and diversity were influenced by environmental factors and host genetic variation during development.

## Introduction

The gastrointestinal tracts of mammals are populated by a dynamic and enormous microbial community that can be viewed as complex, and polygenic traits interacting and coevolving with host genetic and environmental factors (Ley et al., 2006; Sansonetti and Medzhitov, 2009; Yang et al., 2017). The development of metagenomics has allowed for intensive study of the microbial genome and revealed that gut microbiota play important roles in the physiology and immunity of the host (Clemente et al., 2012; Kostic et al., 2015), by enhancing digestive efficiency, promoting immune system development and immune homeostasis, and limiting pathogen colonization (Fraune and Bosch, 2010; Oakley et al., 2014). In mammals, it was previously thought that fetuses lived in a sterile environment and the initial gut microbiota of the infant originated from the mother’s birth canal and body (Tissier, 1900). Recently, more evidence supports the idea that the gut microbiota can be vertically transmitted from the mother to her infant (Blaser, 2006). For example, microorganisms isolated from meconium of healthy newborns by Cesarean section indicated that the microbiota was not derived only postnatally (Jimenez et al., 2008; Moles et al., 2013; Ardissone et al., 2014). Many cultivable microorganisms present in the umbilical cord blood of preterm infants and abundant nonculturable and unclassified microorganisms have been detected in amniotic fluid, suggesting that fetuses are not sterile before delivery (Jimenez et al., 2005; DiGiulio et al., 2008). Morphologic and metagenomic studies have demonstrated that microbes exist in different regions of placenta and microbial DNA can be horizontally transferred from mother to fetus via placenta (Satokari et al., 2009; Stout et al., 2013; Aagaard et al., 2014). Additionally, the construction and succession of gut microbiota were influenced by many factors, such as the mode of delivery, birth environment, and feeding patterns (Dominguez-Bello et al., 2010; Bäckhed et al., 2015).

However, unlike mammals, the avian embryo is an isolated unit whose fertilization occurs *in vivo*, while development occurs in an extracorporeal egg without an umbilical cord, placenta, and amniotic fluid directly associated with the maternal body. The establishment and inheritance of a microbiome in an avian embryo is unknown. The chicken, domesticized from the red jungle fowl, bridges an evolutionary gap between reptiles and mammals, and is an important economical animal that provides humans with meat, eggs, and feathers. It was the first farm animal to have its genome sequenced (International Chicken Genome Sequencing Consortium, 2004). Chickens also serve as a traditional model for studying embryonic development, since its embryos are packaged in eggshells and can be easily observed and manipulated *in vitro* (Brown et al., 2003). Here, we used the chicken as a model organism and performed 16S rRNA sequencing to analyze and compare the microbial composition, abundance, and dynamic distribution during different embryonic stages of development, as well as chicks and their maternal hens (Supplementary Figure S1). In this study, we addressed the following: “(1) Can bacteria be detected in the chicken embryo? (2) If so, can we infer if any of these bacteria are maternally inherited? (3) Are there any core bacterial taxa that are shared across host developmental stages? (4) How does the gut microbiome change during development in a variety of host genetic background?”

## Results

### Profiles of Embryonic Microbiota

**FIGURE 1:**
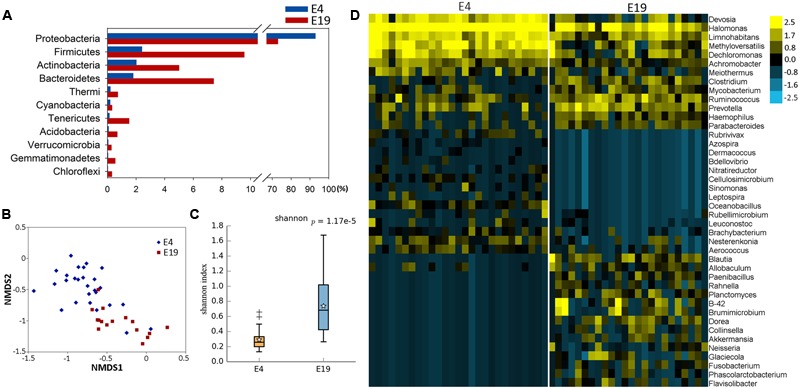
Aggregate microbiota characteristics of 4-day (E4) and 19-day (E19) embryos. **(A)** Dominant taxonomic groups of embryos by phylum. **(B)** Microbial beta diversity of embryos with a Non-metric Multidimensional Scaling (NMDS) plot showing how distant E4 and E19 communities were. **(C)** Microbial alpha diversity with a box plot exhibiting the community diversity (The Shannon estimator). **(D)** Heatmap of hierarchy cluster results for the statistical significant microbial OTUs of two groups at the genus level.

**FIGURE 2:**
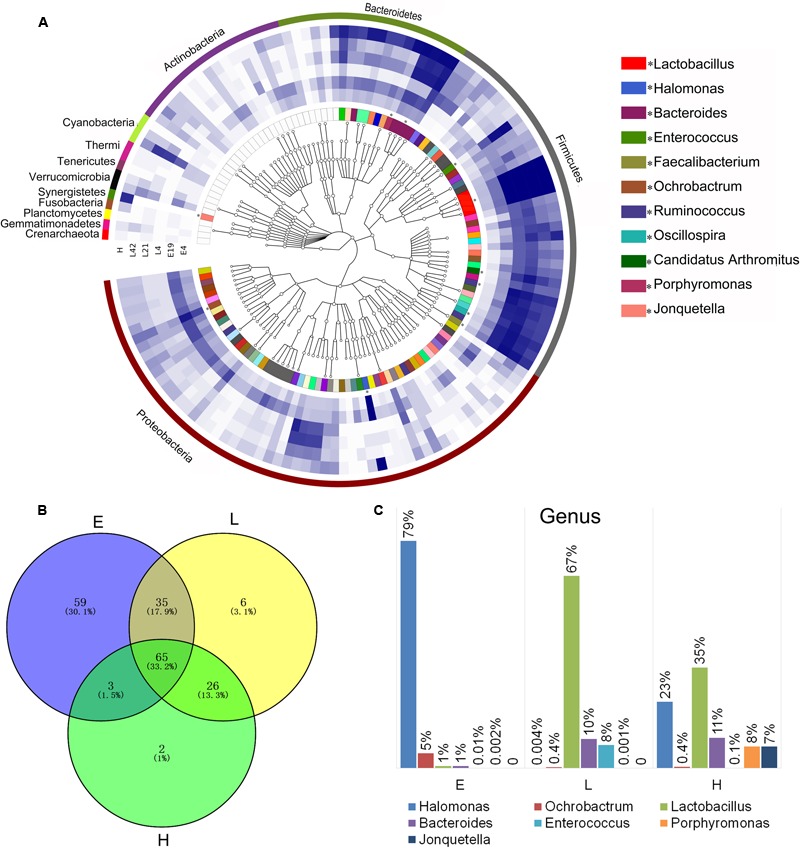
Phylogenetic tree of the taxa and differences in the microbial communities of different development stages. **(A)** Phylogenetic tree constructed from the 188 taxa. Colored blocks in the outermost circle indicate phyla, and in the inner circle indicate genera. The heatmap circles show relative abundance of embryos (E) E4 and E19; chicks (L) at 4 (L4), 21 (L21), and 42 days (L42) post hatch; and maternal hens (H). **(B)** The Venn diagram shows the microbes shared within different host development stages (see Supplementary Table S3 in the Supplementary Material). **(C)** Microbial composition structure of embryos, chicks, and maternal hens at the genus level.

Fifty-one samples, including whole embryos that were incubated for 4 days, and intestines from embryos that were incubated for 19 days, were collected in all breeds. The microbial genomic DNA was isolated from ground embryos or intestines. The microbial classifications revealed that 28 phyla, 162 genera, and 76 species were present in the 4-day and the 19-day embryos (Supplementary Table S1). The most abundant phylum was *Proteobacteria* (86%), followed by *Firmicutes* (5%), *Bacteroidetes* (4%), and *Actinobacteria* (3%) in embryos (Supplementary Figure S2A). The dominant microbial genera were *Halomonas* (79%) and *Ochrobactrum* (5%), which belong to the phylum of *Proteobacteria* (**Figures 1A, 2C**). The correlation coefficient between the microbiota of the 4-day (E4) and the 19-day (E19) embryos was 0.80 (**Table 1**). Although there was similarity of microbial species in phylum between 4-day and the 19-day embryos, we observed a decreasing proportion of *Proteobacteria*, and remarkable increasing tendency of *Firmicutes, Actinobacteria*, and *Bacteroidetes* (**Figure 1A**). Accordingly, *Halomonas* was significantly different between the two groups, which were 88% and 55%, respectively. Microbial beta diversity of embryos with a Non-metric Multidimensional Scaling (NMDS; unweighted UniFrac distance) plot and heatmap showed the characteristics of the 4-day and 19-day indicating a significant different of microbial communities (**Figures 1B,D**). Signature genera at 4-day included bacteria found in 19-day, i.e., *Halomonas, Bacteroidetes*, and *Ochrobactrum*, in addition, alpha diversity analysis revealed more microbial diversity in the 19-day embryos than the 4-day embryos (**Figures 1C, 2A** and Supplementary Figure S3).

**Table 1:** Gut microbial correlation coefficients at different host development stages.

Group	E4	E19	L4	L21	L42	H
E4	–	0.852	0.637	0.440	0.477	0.412
E19	0.799	–	0.625	0.458	0.492	0.393
L4	0.502	0.442	–	0.835	0.801	0.577
L21	0.261	0.219	0.765	–	0.896	0.716
L42	0.325	0.259	0.686	0.797	–	0.653
H	0.364	0.300	0.578	0.642	0.670	–

*Upper side triangle is the microbial correlation at the family level; lower side triangle is the microbial correlation at the genus level.*

### Composition of Gut Microbiome in Different Host Developmental Stages

In order to investigate the establishment and inheritance of gut microbiota during host development, we compared in three breeds the composition and abundance of gut microbiota in maternal hens (H), embryos (E) E4 and E19, and chicks (L) at 4 (L4), 21 (L21), and 42 days (L42) post hatch (**Figure 2A** and Supplementary Figure S1). One thousand seven hundred and nine microbial operational taxonomic units (OTUs) were detected in at least 75% of the samples, among them, 33 phyla and 196 genera of gut microbes were annotated (Supplementary Table S2). The most abundant phyla in all stages were *Proteobacteria, Firmicutes*, and *Bacteroidetes*, followed by *Actinobacteria, Cyanobacteria*, and *Synergistetes* (**Figure 2A**), which were consistent with previous studies (Waite and Taylor, 2014). However, the proportion of these phyla throughout the stages was different. *Proteobacteria* was mainly present in embryos (86%) and maternal hens (22%). *Firmicutes* (76% and 44%) and *Bacteroidetes* (10% and 24%) were the two major phyla in chicks and maternal hens, respectively (Supplementary Figure S2A). At the genus level, *Halomonas* was the dominant microbe in embryos (79%), and *Lactobacillus* (67%) and *Bacteroides* (10%) were the most abundant microbes in chicks (**Figure 2C**). Fifty-nine genera of microbes with low abundance were detected only in embryos. The declining population of those microbes during embryonic development and an absence in chicks and maternal hens suggested that some microbes were temporarily harbored in embryos and they were influenced by genetic and environmental factors during host development (**Figures 2A,B** and Supplementary Table S3). Abundance of *Enterococcus, Ruminococcus*, and *Oscillospira* was high in chicks and low in embryos and maternal hens. *Jonquetella, Sneathia*, and *Porphyromonas* whose function related to physiological and environmental adaptation (Jumas-Bilak et al., 2012; Belibasakis et al., 2013) showed abundant proportions in maternal hens (**Figure 2A**). Among the 162 genera that were detected in embryos, 68 and 100 of them were detected in maternal hens and chicks, respectively. The correlation coefficients for microbiota in embryos and maternal hens were approximately 0.33 and 0.40 at the genus and family level, respectively, and for embryos and chicks, it decreased initially and subsequently increased during the development of host stages (**Table 1**) with the correlation coefficient of 0.52 at the family level. Of the132 genera detected in chicks 91 were also observed in the maternal hens (**Figure 2B**). Correlation coefficients ranging from 0.58 to 0.72 between chicks and maternal hens were found at the family level (**Table 1**). Principle component analysis (PCA) indicated that the gut microbial composition in embryos was different from that in chicks and maternal hens, while similar between chicks and maternal hens (Supplementary Figure S2B). These results support the hypothesis that early colonizers of embryos were inherited from their maternal hens and a transmission process of gut microbiota from maternal hen to embryo and embryo to chick exists. The higher microbial diversity in embryos than in chicks and maternal hens indicates that the microbial diversity declined during host development, and provides additional evidence for the hypothesis that the harbored microbiome is influenced by the host during development (**Figure 2A** and Supplementary Figure S2C).

**FIGURE 3:**
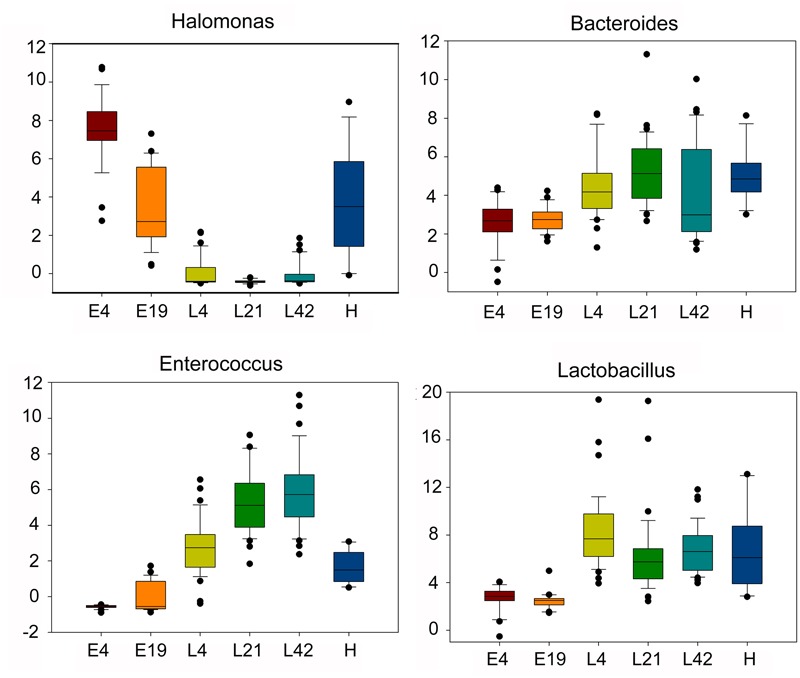
The dynamic distribution of main core microbes within different host development stages including embryos (E) E4 and E19; chicks (L) at 4 (L4), 21 (L21), and 42 days (L42) post hatch; and maternal hens (H) based on microbial abundance. The boxes have lines at the lower quartile, median, and upper quartile values.

Among 196 annotated genera, 65 were considered as core microbes which existed in embryos, chicks, and maternal hens (**Figures 2A,B** and **Table 2**), such as *Halomonas, Lactobacillus, Bacteroides*, and *Enterococcus* (**Figure 3**). *Halomonas* had a high abundance in embryos and maternal hens, with a decreasing trend in chicks. This suggested that it may be an essential microbe and it may be susceptible to environmental effects. *Lactobacillus* and *Enterococcus* exhibited an increasing trend from embryo to chick and stabilized in maternal hens. *Bacteroides* remained steady in all stages (**Figure 3**). These results suggest that most of the microbial colonizers harbored in early embryos may originate from the maternal hens, and can be influenced by host genetic and environmental factors during developmental stages.

**Table 2:** Sixty-five core microbes which were common to embryo, chick, and maternal hen in Venn diagram.

Phylum	Genus	Phylum	Genus
Actinobacteria	*Amycolatopsis*	Firmicutes	*Turicibacter*
Actinobacteria	*Bifidobacterium*	Firmicutes	*Veillonella*
Actinobacteria	*Bradyrhizobium*	Fusobacteria	*Fusobacterium*
Actinobacteria	*Brevibacterium*	Proteobacteria	*Acinetobacter*
Actinobacteria	*Collinsella*	Proteobacteria	*Aminobacter*
Actinobacteria	*Corynebacterium*	Proteobacteria	*Burkholderia*
Actinobacteria	*Rhodococcus*	Proteobacteria	*Dechloromonas*
Actinobacteria	*Streptomyces*	Proteobacteria	*Desulfovibrio*
Bacteroidetes	*Bacteroides*	Proteobacteria	*Desulfuromonas*
Bacteroidetes	*Flavisolibacter*	Proteobacteria	*Enhydrobacter*
Bacteroidetes	*Flavobacterium*	Proteobacteria	*Erwinia*
Bacteroidetes	*Parabacteroides*	Proteobacteria	*Haemophilus*
Bacteroidetes	*Pedobacter*	Proteobacteria	*Halomonas*
Bacteroidetes	*Porphyromonas*	Proteobacteria	*Hyphomicrobium*
Bacteroidetes	*Prevotella*	Proteobacteria	*Klebsiella*
Bacteroidetes	*Sediminibacterium*	Proteobacteria	*Limnohabitans*
Euryarchaeota	*Methanobrevibacter*	Proteobacteria	*Marinobacter*
Firmicutes	*Anaerostipes*	Proteobacteria	*Methyloversatilis*
Firmicutes	*Anoxybacillus*	Proteobacteria	*Mycoplana*
Firmicutes	*Blautia*	Proteobacteria	*Ochrobactrum*
Firmicutes	*Butyricimonas*	Proteobacteria	*Phenylobacterium*
Firmicutes	*Clostridium*	Proteobacteria	*Phyllobacterium*
Firmicutes	*Coprococcus*	Proteobacteria	*Pseudomonas*
Firmicutes	*Dorea*	Proteobacteria	*Rhizobium*
Firmicutes	*Enterococcus*	Proteobacteria	*Rhodobacter*
Firmicutes	*Eubacterium*	Proteobacteria	*Sphingomonas*
Firmicutes	*Faecalibacterium*	Proteobacteria	*Sutterella*
Firmicutes	*Lactobacillus*	Proteobacteria	*Thiobacillus*
Firmicutes	*Lactococcus*	Proteobacteria	*Zoogloea*
Firmicutes	*Megamonas*	Thermi	*Thermus*
Firmicutes	*Oscillospira*	Verrucomicrobia	*Prosthecobacter*
Firmicutes	*Ruminococcus*	Verrucomicrobia	*Akkermansia*
Firmicutes	*Staphylococcus*		

### Functional Performance of Microbiota in Different Host Developmental Stages

**FIGURE 4:**
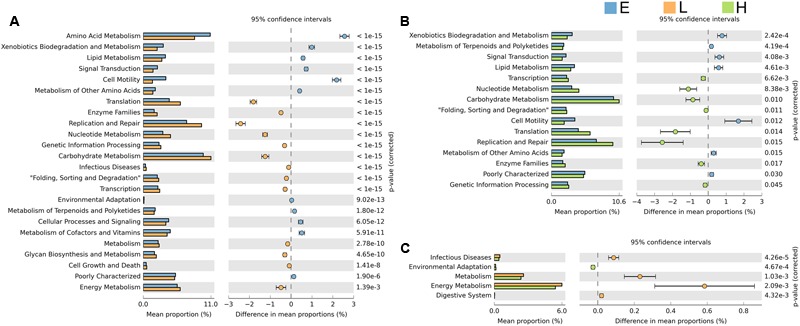
Significant differences in microbial metabolic pathways at different stages. Extended error bar plot indicates the difference in mean proportion of microbial metabolic pathways between the two groups along with the associated confidence interval of this effect size and the *p*-value of Welch’s *t*-test (*p* < 0.05). **(A)** Embryo vs. chick. **(B)** Embryo vs. maternal hen. **(C)** Chick vs. maternal hen.

Comparing the functional capacity of the gut microbiota, in different host developmental stages including all breeds, could help us understand the relationships between the gut microbiota and its host. Twenty-nine abundant secondary metabolism pathways were identified while 25 pathways showed significant differences among embryos, chicks, and maternal hens in all breeds (Supplementary Table S4). As the gut microbiota of embryos evolve, they may develop more complex functional pathways such as substance metabolism, cell motility, signal transduction, cellular processes and signaling, cofactors, and vitamins metabolism. Most of these pathways are related to growth and development. Pathways allocated for translation, replication and repair, nucleotide metabolism, genetic information processing, carbohydrate metabolism, and transcription were more enriched in the microbiome harbored in chicks and maternal hens (**Figure 4**). The microbiota in chicks have a high abundance of functional capacities involved in metabolic pathways such as infection diseases, energy metabolism, and the digestive system (**Figure 4C**). Fewer differences in metabolism pathways between chicks and maternal hens indicated that, although they have a different gut microbial composition, their gut microbiome performs similar functions. Photosynthesis-antenna proteins usually exist in phycobilisomes of *cyanobacteria* and act as peripheral antenna systems enabling more efficient absorption of light energy. In our study, photosynthesis-antenna proteins, which fall into the category of energy metabolism, were enriched in embryos but not in chicks nor maternal hens (Supplementary Figure S4). Calcium signaling pathways are known to participate in bone formation and brain development in fetuses and in increasing bone mineral content of elders (Maas et al., 2005; Chen et al., 2010). We believe that the high abundance of microorganisms with calcium signaling pathways in the microbiota from embryos may be associated with growth and development. Microbiota whose function related to the replication and repair systems, such as nucleotide excision repair and non-homologous end-joining, which are involved in DNA repair to resist infectivity damage, were significantly enriched in chicks and maternal hens. The phosphotransferase system (PTS) is a major mechanism used by bacteria for uptake of carbohydrates, particularly hexoses, hexitols, and disaccharides, to enable the use of phosphoenolpyruvate as a source of energy. Microbiota related to this system were also enriched in chicks and maternal hens (Supplementary Figure S4). This phenomenon may be caused by the food intake of chicks and maternal hens and suggest that there is an inevitable relationship between growth and development of the host and microbial functional capacity.

### Host Genetic Background Influences the Gut Microbiota

**FIGURE 5:**
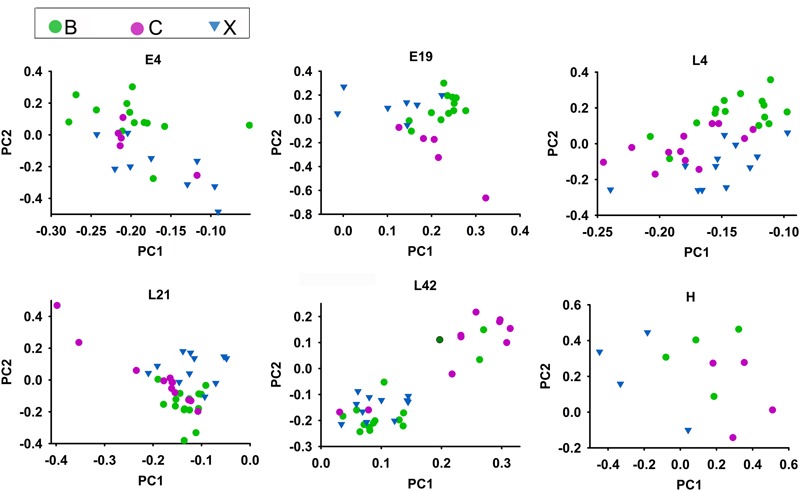
Microbiota compositions for Beijing Fatty (B), Shiqiza (C), and Xianju (X) of chickens within different host development stages including embryos (E) E4 and E19; chicks (L) at 4 (L4), 21 (L21), and 42 days (L42) post hatch; and maternal hens (H).

In the above results, microbial community and functional capacity were analyzed in different host developmental stages including all breeds. To investigate the extent of the influence of host genetic factors on gut microbiota, we also studied microbial composition and abundance in three native Chinese breeds: Beijing Fatty (B), Shiqiza (C), and Xianju (X) chickens. 16S rRNA annotation results revealed that, under similar feeding conditions, the three breeds had similar dominant microorganism communities at the genus level: *Lactobacillus, Halomonas, Bacteroides*, and *Enterococcus* (Supplementary Figures S5A,B). Alpha diversity results indicated that there was no difference in the richness and diversity of the gut microbiota among the Beijing Fatty, Shiqiza, and Xianju chickens (Supplementary Figure S5D). Ninety-four percent genera (185/196) from the gut microbiota were common in Beijing Fatty, Shiqiza, and Xianju chickens (Supplementary Figure S5C). Although the dominant microorganisms were uniform among the breeds, 109 genera were significantly different (*p* < 0.05) among Beijing Fatty, Shiqiza, and Xianju chickens (Supplementary Table S5). Principal component analysis showed that the samples clustered according to breeds within different host developmental stages, and demonstrated that the Beijing Fatty, Shiqiza, and Xianju separated clear in E4 and E19 embryos (**Figure 5**). However, these three breeds were more dispersed and similar in chicks and maternal hens compared to embryos indicating the similar feeding conditions influence the construction of host microbiota (**Figure 5**). The microbiota were still influenced by breed, albeit with more similarities in chicks and maternal hens.

## Discussion

Studies of gut microbial establishment have been performed most in adult animals or newborn offspring, such as human neonates (Mackie et al., 1999), insects (Tang et al., 2012), zebra fish (Davis et al., 2016), chickens (Ding et al., 2016), snakes (Costello et al., 2010; McLaughlin et al., 2015), alligators (Keenan et al., 2013; Keenan and Elsey, 2015), and lizards (Ren et al., 2016; Kohl et al., 2017), while few studies involved the fetus or embryo. In our study, microbiota were detected qualitatively in the embryo. The formation of the egg is an open process where large amounts of lipids and proteins are transported to the oocyte in the maternal hen ovary. When the oocyte is ovulated, fertilization occurs in the funnel of the infundibulum, and then the oosperm is packaged with secretory proteins in the oviduct to form a fertilized egg. Although there is no umbilical cord, placenta, or amniotic fluid directly associated with the maternal hen, avian embryos obtain abundant nutrients from maternal hen. Mammalian studies have suggested that the upper female reproductive tract is an essential place for the beginning of life. In a recent study, researchers found different types of bacteria in the ovaries and fallopian tubes which suggested that the upper female reproductive tract was not free from microorganisms (Keku, 2016). Additional evidence indicated that vaginal tract contains a remarkably complex microbial community which can be transmitted from mother to fetus (Mackie et al., 1999; Schultz et al., 2004; White et al., 2011; Martin, 2012; Prince et al., 2014). The oviduct of chickens joins the urinary and digestive tracts in the cloaca which may result in the transmission of microbiota from the cloacae through the oviduct and even the body cavity (Gross and Siegel, 1959; Stanley et al., 2015; Zhao et al., 2015; Pedroso et al., 2016). So, it is a normal phenomenon to detect microbes in the embryo. Also, there is possible that the microbes were from the environment, chemical reagent, or the process of sample collection (Salter et al., 2014). However, in order to verify whether the microbiota were contaminated by the environment or experimenter, we performed the preliminary experiments. Seven embryos were selected as the experimental group (B) and five embryo-free samples as the control group (C) for 16S rRNA sequencing. Comparing the microbial OTUs between the two groups, we found that there are much more abundant microbial taxa (Supplementary Figure S6A) in the experimental than the control group. The result of rarefaction curve also indicated the deficient sequencing depth in control group (Supplementary Figure S6B). Further analysis revealed significant differences in microbial diversity and abundance between the experimental and control groups (Supplementary Figure S6C). As suggested above, we speculated that the establishment and inheritance of the embryo gut microbiota were derived from the maternal hen by the process of fertilization and egg formation in the oviduct. Further studies are still needed to determine the microbial composition of avian reproductive system.

In order to adapt to their rapidly changing developmental environments, patterns of gut microbial diversity and abundance varied across different host development stages. However, some of the gut microbial membership in embryos, chicks, and maternal hens was strikingly similar, with a shared core microbiota. The definition of core microbiota has been addressed in fish and mammals (Turnbaugh et al., 2009; Roeselers et al., 2011; Tang et al., 2012), although the producing mechanisms of core microbiota remain unclear. Our study suggests that core gut microbiota also exists in chickens where 65 genera were detected and considered as core microbes in embryos, chicks, and maternal hens. Interestingly, *Halomonas*, which belongs to *Halomonadaceae* family and *Proteobacteria* phylum, was the dominant genus in embryos and maternal hens but not chicks (**Figure 2A**). *Halomonadaceae* are Gram-negative, chemoorganotrophic, aerobic, or facultative anaerobic, and moderately halophilic, haloalkaliphilic, halotolerant, or non-halophilic. Certain *Halomonas* species are also known to have a temperature-dependent salt requirement or tolerance and are able to utilize cellulose as carbon sources, although Borsodi et al. (2005) did not observe direct cellulolytic activity. *Halomonas venusta* was identified as the abundant species in the embryos and maternal hens. Berlanga et al. (2014) reported that *H. venusta* retained the capacity of sessile cells to adhere to polystyrene and form a biofilm. Ectoine, which is synthesized and released by *H. venusta*, can balance the cell’s osmotic pressure and protect the structures of enzymes, DNA, and the cytomembrane, implying that *H. venusta* may be involved in cell motility, amino acid, carbohydrate metabolism, and environmental adaptation in embryos and maternal hens (Lippert and Galinski, 1992; Zheng et al., 2010; Zhou et al., 2014). A strong correlation between bacterial growth and microbial utilization of phenol was discovered, and *H. venusta* was able to consume phenol as the sole carbon source indicating it can degrade toxic and recalcitrant compounds (Munoz et al., 2001). Compared with chicks and maternal hens, xenobiotics biodegradation and metabolism were significantly enriched in embryos of our study (**Figures 4A,B** and Supplementary Figure S7). As embryos produce poisonous and harmful substances during development, we speculate that the existence of *H. venusta* can assist the host by decomposing these toxic compounds. Therefore, the abundance of specific genera within the chicken core microbiota may be due, in part, to the host distinct growth and development, as well as diet in each developmental stage, i.e., the transition from embryos to chicks which utilizing the external feeds that are rich in carbohydrates (Noy et al., 2001; Zhao et al., 2013). Our results provide evidence for the hypothesis that the composition of embryo microbiome was inherited part from maternal hens and adjusted by host genetic and environmental factors during different developmental stages.

## Materials and Methods

### Study Design and Animal Sampling

The intent of this study was to characterize the gut microbial establishment and inheritance from maternal hen to embryo and to chick (Supplementary Figure S1). We selected 12 individual maternal hens at 35 weeks of age from three breeds (Beijing Fatty, Xianju, and Shiqiza) from the Animal Husbandry and Veterinary Research Institute in the Shanghai Academy of Agricultural Science for reproduction. Fertilized eggs from the selected maternal hens were used to hatch embryos and chicks which also include three different breeds (Supplementary Figure S1). The incubation was performed in Ova-Easy Advance Series II Digital Cabinet Egg Incubator (Brinsea, United Kingdom) within a sterilized room at 37.8°C and 55–65% humidity. To investigate embryo gut microbiota, we aseptically collected 51 samples including whole embryos that had incubated to day 4, and intestines from embryos that had incubated to day 19. The collected embryo samples were immediately frozen at -80°C. The procedures of embryonic sample collection and subsequent operation were carried out on a clean bench under aseptic conditions. The newly hatched chicks were maintained in same husbandry conditions with their maternal hen. Fecal samples from the maternal hens (*n* = 12) at reproduction and chicks (*n* = 113) aged 4, 21, and 42 days were collected (Supplementary Table S6). All of the fecal specimens were kept on ice when collecting, transported to the laboratory and frozen at -80°C immediately until further analysis. The procedures of embryonic sample collection, microbial genomic DNA extraction, and PCR amplification were performed with sterile procedures. Protocols used for this experiment were approved by ethics committee for the Care and Use of Laboratory Animals in Shanghai Jiao Tong University, China.

### DNA Extraction and 16S rRNA Sequencing

Genomic DNA was isolated from intestinal and fecal samples using TIANGEN DNA Stool Mini Kit (TIANGEN, cat#DP328) following the manufacturer’s instructions. Harvested DNA was quantified on a Nanodrop spectrophotometer (Thermo scientific, United States) to assess the quantity and quality of the DNA. The V4 hypervariable region of the 16S rRNA gene was amplified by PCR using sample-specific sequence barcoded fusion primers: forward 5′-AYTGGGYDTAAAGNG-3′, reverse 5′-TACNVGGGTATCTAATCC-3′. The PCR reaction conditions were: 94°C for 5 min; 94°C for 30 s, 50°C for 30 s, and 72°C for extension; repeated for 27 cycles; with a final 72°C for 7 min (Zhao et al., 2013). The PCR products were excised from a 1.5% agarose gel and purified using a QIAGEN quick Gel Extraction Kit (QIAGEN, cat#28706). Purified PCR products from 176 samples were used to construct a sequencing library using Illumina TruSeq (Illumina, United States) following the manufacturer’s suggested protocols. 16S rRNA sequencing was performed at the Shanghai Personal Biotechnology Limited Company, Shanghai, China, using the Illumina MiSeq (Illumina, United States) sequencing platform. Barcoded V4 amplicons were sequenced using the pair-end method by Illumina Miseq. Original pair-end sequences with mean quality lower than 30, containing ambiguous bases, sequence length shorter than 150 bp, chimera, adaptor contamination, or host contaminating reads were removed. The original pair-end sequence reads that passed our quality control criteria and contained a sequence overlap of at least 10 bp without any mismatch were assembled according to their overlap sequence. A total of 9,887,520 sequences from the V4 region of the 16S rRNA sequence from 176 samples that passed our quality filters were used for this study (Supplementary Table S7), with an average length of 225 bp for each sequence (Supplementary Figure S8). Trimmed sequences were uploaded to QIIME for further analysis. The metagenome sequences used in this paper are publicly available from Metagenome Rapid Annotation using Subsystem Technology^1^.

### Taxonomic Assignment and Statistical Analysis

Microbial OTUs were derived from the trimmed sequences of the PCR amplicon for the V4 hypervariable region of the 16S rRNA gene which were compared to the Greengene (DeSantis et al., 2006) databases using the uclust and blast functions in QIIME (Caporaso et al., 2010). 76,840 OTUs were annotated from the 9,887,520 amplicons and classified at the domain, phylum, class, order, family, genus, and species levels (Supplementary Table S8). Then, OTUs that were present in at least 44 samples were used for statistical analysis. The OTU abundance count was log2 transformed and then normalized as follows: from each log-transformed measure, the arithmetic mean of all transformed values was subtracted; the difference was divided by the standard deviation of all log-transformed values for the given sample. After this procedure, the abundance profiles for all samples exhibited a mean of 0 and a standard deviation of 1 (Zhao et al., 2013). Normalized abundance was used to generate a heatmap by Cluster3.0 and Java Treeview (Eisen, 2002) and to draw a 3dPCA figure by R (Dessau and Pipper, 2008). Alpha-diversity analysis was performed in maternal hens (Schloss et al., 2009) with the alpha-diversity.py script to calculate the chao1 (Pitta et al., 2014) and Shannon (Mahaffee and Kloepper, 1997) metrics. The Venn diagrams were generated using Venny (Oliveros, 2007). Box plots and bar charts were performed by SigmaPlot (Kornbrot, 2000) and STAMP (Parks and Beiko, 2010), and all *p*-values were adjusted by the Benjamini–Hochberg FDR procedure (Benjamini and Hochberg, 1995). The phylogenetic tree was generated by GraPhlAn, which is a software tool for producing high-quality circular representations of taxonomic and phylogenetic trees (Asnicar et al., 2015). Correlation analysis was conducted to identify the association of microbiome among different developmental stages using Microsoft Excel (Pearson, 1901).

### Functional Profile Analysis

Microbial functional profile was predicted using PICRUSt (Langille et al., 2013). The OTUs were mapped to gg13.5 database at 97% similarity by QIIME’s command “pick_closed_otus.” The OTU abundance was normalized automatically using 16S rRNA gene copy numbers from known bacterial genomes in Integrated Microbial Genomes (IMG). The predicted genes and their functions were aligned to Kyoto Encyclopedia of Genes and Genomes (KEGG) (Kanehisa et al., 2014) database and the differences among groups were compared using the software STAMP. Two-sided Welch’s *t*-test (Welch, 1947) and Benjamini–Hochberg FDR correction were used in group analysis.

## Ethics Statement

Protocols used for this experiment were approved by ethics committee for the Care and Use of Laboratory Animals in Shanghai Jiao Tong University, China.

## Author Contributions

JD, YZ, and HM wrote the manuscript. JD and HM conceived and designed the experimental procedure, and supervised the study. JD performed statistical analysis, sample collection, DNA extraction, and data processing. RD, LY, and CH participated in sample collection, statistical analysis, and figure design. KX, LX, and LL coordinated sample collection. All authors read and approved the final manuscript.

## Conflict of Interest Statement

The authors declare that the research was conducted in the absence of any commercial or financial relationships that could be construed as a potential conflict of interest.

## Footnotes

1 http://metagenomics.anl.gov/mgmain.html?mgpage=project&project=20686
