# Supplementary material for: Inheritance and Establishment of Gut Microbiota in Chickens
Source: Front Microbiol. 2017 Oct 10;8:1967. doi: 10.3389/fmicb.2017.01967 (PMC5641346; doi:10.3389/fmicb.2017.01967)
Supplement: Supplementary file 1 [file Table_1.DOCX]

**Supplemental Tables**

**Table S1-S8**

**TABLE S1** Microbes of embryo in different levels

| Level | Phylum | Class | Order | Family | Genus | Species |
| --- | --- | --- | --- | --- | --- | --- |
| Number | 28 | 33 | 51 | 86 | 162 | 76 |

**TABLE S2** Microbes of all samples (embryo, chick and maternal hen) in different levels.

| Level | Phylum | Class | Order | Family | Genus | Species |
| --- | --- | --- | --- | --- | --- | --- |
| Number | 33 | 77 | 117 | 171 | 196 | 110 |

**TABLE S3** Abundance of microbiota in embryo (E), chick (L) and maternal hen (H) (Related to Figure 2B).

| **Number of microbiota** | **Genus** | **Summed relative abundance** | **Genus** | **Summed relative abundance** |
| --- | --- | --- | --- | --- |
| 65 core taxons in "E", "L" and "H": | Acinetobacter | 23827 | Lactobacillus | 3558665 |
|  | Akkermansia | 1510 | Lactococcus | 8120 |
|  | Aminobacter | 848 | Limnohabitans | 5983 |
|  | Amycolatopsis | 7793 | Marinobacter | 11177 |
|  | Anaerostipes | 586 | Megamonas | 5077 |
|  | Anoxybacillus | 1720 | Methanobrevibacter | 181 |
|  | Bacteroides | 583606 | Methyloversatilis | 43843 |
|  | Bifidobacterium | 10134 | Mycoplana | 1165 |
|  | Blautia | 5885 | Ochrobactrum | 119548 |
|  | Bradyrhizobium | 6249 | Oscillospira | 97028 |
|  | Brevibacterium | 1741 | Parabacteroides | 5978 |
|  | Burkholderia | 1969 | Pedobacter | 5869 |
|  | Butyricimonas | 1213 | Phenylobacterium | 4169 |
|  | Clostridium | 20250 | Phyllobacterium | 1838 |
|  | Collinsella | 363 | Porphyromonas | 47659 |
|  | Coprococcus | 21999 | Prevotella | 13879 |
|  | Corynebacterium | 6751 | Prosthecobacter | 2313 |
|  | Dechloromonas | 7198 | Pseudomonas | 17328 |
|  | Desulfovibrio | 2575 | Rhizobium | 1251 |
|  | Desulfuromonas | 31845 | Rhodobacter | 498 |
|  | Dorea | 10403 | Rhodococcus | 31172 |
|  | Enhydrobacter | 1187 | Ruminococcus | 109736 |
|  | Enterococcus | 397896 | Sediminibacterium | 4162 |
|  | Erwinia | 498 | Sphingomonas | 17682 |
|  | Eubacterium | 592 | Staphylococcus | 18872 |
|  | Faecalibacterium | 120352 | Streptomyces | 17834 |
|  | Flavisolibacter | 338 | Sutterella | 3027 |
|  | Flavobacterium | 9891 | Thermus | 4956 |
|  | Fusobacterium | 1752 | Thiobacillus | 3654 |
|  | Haemophilus | 1423 | Turicibacter | 14940 |
|  | Halomonas | 1651563 | Veillonella | 2067 |
|  | Hyphomicrobium | 450 | Zoogloea | 7382 |
|  | Klebsiella | 40775 |  |  |
| 35 taxon detected in "E" and "L": | Acetobacter | 271 | Mesorhizobium | 306 |
|  | Achromobacter | 1191 | Methylobacterium | 2411 |
|  | Aerococcus | 630 | Natronincola_Anaerovirgula | 1544 |
|  | Agrobacterium | 6697 | Neisseria | 234 |
|  | Alistipes | 182 | Paracoccus | 672 |
|  | Allobaculum | 6242 | Planctomyces | 624 |
|  | Arthrobacter | 1103 | Propionibacterium | 265 |
|  | Asticcacaulis | 201 | Pseudoramibacter_Eubacterium | 493 |
|  | Bacillus | 10412 | Pseudoxanthomonas | 231 |
|  | Brachybacterium | 390 | Rahnella | 383 |
|  | Brevundimonas | 921 | Ralstonia | 1104 |
|  | Cellvibrio | 196 | Rhodoplanes | 581 |
|  | Chryseobacterium | 708 | Sphingobacterium | 259 |
|  | Desulfococcus | 5534 | Sphingobium | 1903 |
|  | Kribbella | 367 | Sphingopyxis | 1240 |
|  | Kuenenia | 374 | Streptococcus | 7477 |
|  | Leuconostoc | 255 | Sulcia | 1620 |
|  | Meiothermus | 755 |  |  |
| 26 taxon detected in "L" and "H": | Aeriscardovia | 11507 | Dehalobacterium | 675 |
|  | AF12 | 6679 | Gallibacterium | 5824 |
|  | Anaerotruncus | 565 | Helicobacter | 8961 |
|  | Barnesiella | 6466 | Holdemania | 417 |
|  | Bilophila | 3086 | Methanocorpusculum | 133 |
|  | Blvii28 | 626 | Mucispirillum | 430 |
|  | Bulleidia | 1013 | Odoribacter | 6118 |
|  | Butyricicoccus | 8062 | Paludibacter | 206 |
|  | Campylobacter | 49920 | Peptococcus | 5498 |
|  | Candidatus_Arthromitus | 79936 | Rothia | 6270 |
|  | Candidatus_Azobacteroides | 187 | Vestibaculum | 211 |
|  | cc_115 | 17035 | Wautersiella | 177 |
|  | Coprobacillus | 14654 | YRC22 | 544 |
| 3 taxon detected in "E" and "H": | Actinomyces | 2940 | Paraprevotella | 220 |
|  | Sulfurimonas | 192 |  |  |
| 59 taxon only detected in "E": | Actinomadura | 867 | Leadbetterella | 1273 |
|  | Adhaeribacter | 208 | Luteimonas | 992 |
|  | Aequorivita | 905 | Luteolibacter | 244 |
|  | Alcanivorax | 1186 | Methylomonas | 583 |
|  | B-42 | 2878 | Micrococcus | 299 |
|  | Brumimicrobium | 1798 | Mycobacterium | 850 |
|  | Candidatu_Solibacter | 272 | Nesterenkonia | 315 |
|  | Candidatus_Hepatoplasma | 961 | Nocardioides | 607 |
|  | Candidatus_Rhabdochlamydia | 183 | Novosphingobium | 417 |
|  | Carnobacterium | 269 | Oceanobacillus | 246 |
|  | Coprothermobacter | 387 | Paenibacillus | 334 |
|  | DA101 | 964 | Pandoraea | 136 |
|  | Deinococcus | 581 | Phascolarctobacterium | 247 |
|  | Desulfosarcina | 673 | Planomicrobium | 307 |
|  | Desulfosporosinus | 234 | Pseudidiomarina | 789 |
|  | Dethiosulfatibacter | 2248 | Pseudoalteromonas | 513 |
|  | Devosia | 3410 | Pseudogulbenkiania | 161 |
|  | Dokdonella | 193 | Rhodanobacter | 2444 |
|  | Dyadobacter | 208 | Rubellimicrobium | 154 |
|  | Dysgonomonas | 457 | Salinibacterium | 180 |
|  | Emticicia | 162 | Salinispora | 324 |
|  | Georgenia | 284 | Segetibacter | 191 |
|  | Glaciecola | 229 | Shewanella | 855 |
|  | GOUTA19 | 151 | Solibacillus | 428 |
|  | Hymenobacter | 289 | Stenotrophomonas | 171 |
|  | Hyphomonas | 305 | Sulfuritalea | 282 |
|  | Kaistobacter | 513 | T78 | 845 |
|  | Kosmotoga | 482 | Thermobifida | 646 |
|  | Labrys | 180 | Vibrio | 306 |
|  | Lachnospira | 328 |  |  |
| 6 taxon only detected in "L": | Eggerthella | 1613 | Brevibacillus | 367 |
|  | Proteus | 1276 | Facklamia | 177 |
|  | Weissella | 408 | CF231 | 171 |
| 2 taxon only detected in "H": | Jonquetella | 47323 | Sneathia | 260 |

**TABLE S4** Functional maturation among different stages (Related to Figure 4).

| KEGG metabolism pathway | P-values (after FDR corrected) (*p<0.05,**p<0.01) | | |
| --- | --- | --- | --- |
|  | E vs L | E vs H | L vs H |
| Amino Acid Metabolism | 0.0000** | 0.1313 | 10.7167 |
| Biosynthesis of Other Secondary Metabolites | 3.6398 | 4.2597 | 1.2521 |
| Carbohydrate Metabolism | 0.0000** | 0.0102* | 1.8179 |
| Cell Growth and Death | 0.0000** | 1.0363 | 7.0089 |
| Cell Motility | 0.0000** | 0.0115* | 5.1480 |
| Cellular Processes and Signaling | 0.0000** | 0.8956 | 10.8517 |
| Digestive System | 0.3895 | 1.2768 | 0.0043** |
| Energy Metabolism | 0.0014** | 10.0609 | 0.0021** |
| Environmental Adaptation | 0.0000** | 11.9491 | 0.0005** |
| Enzyme Families | 0.0000** | 0.0169* | 7.8600 |
| Folding Sorting and Degradation | 0.0000** | 0.0108* | 0.0742 |
| Genetic Information Processing | 0.0000** | 0.0448* | 0.2262 |
| Glycan Biosynthesis and Metabolism | 0.0000** | 2.5391 | 4.1870 |
| Immune System | 13.1573 | 12.9456 | 9.4475 |
| Infectious Diseases | 0.0000** | 0.8038 | 0.0000** |
| Lipid Metabolism | 0.0000** | 0.0046** | 27.4134 |
| Membrane Transport | 0.3473 | 17.9478 | 9.8613 |
| Metabolism | 0.0000** | 3.1544 | 0.0010 |
| Metabolism of Cofactors and Vitamins | 0.0000** | 0.8524 | 26.9491 |
| Metabolism of Other Amino Acids | 0.0000** | 0.0151* | 4.2011 |
| Metabolism of Terpenoids and Polyketides | 0.0000** | 0.0004* | 20.9195 |
| Nervous System | 0.3997 | 13.0483 | 15.6954 |
| Nucleotide Metabolism | 0.0000** | 0.0084** | 16.1610 |
| Poorly Characterized | 0.0000** | 0.0305* | 6.3377 |
| Replication and Repair | 0.0000** | 0.0151* | 22.8940 |
| Signal Transduction | 0.0000** | 0.0041** | 11.7718 |
| Transcription | 0.0000** | 0.0066** | 24.4348 |
| Translation | 0.0000** | 0.0140* | 26.9560 |
| Xenobiotics Biodegradation and Metabolism | 0.0000** | 0.0002** | 2.5399 |

**TABLE S5** The comparison of genus abundance of Beijing Fatty (B), Xianju (X) and Shiqiza (C) chickens in different stages.

| GENUS | | P-value（after FDR corrected）(*p<0.05,**p<0.01) | | |
| --- | --- | --- | --- | --- |
|  |  | B vs C | B vs X | C vs X |
| H | Streptomyces | 0.867 | 0.042* | 0.015* |
| E4 | Mycobacterium | 0.843 | 0.048* | 0.301 |
|  | Chryseobacterium | 0.518 | 0.033* | 0.408 |
|  | Burkholderia | 0.518 | 0.055 | 0.015* |
| E19 | Amycolatopsis | 0.895 | 0.074 | 0.081 |
|  | Phyllobacterium | 0.518 | 0.127 | 0.042* |
|  | Burkholderia | 0.293 | 0.240 | 0.039* |
|  | Aminobacter | 0.843 | 0.033* | 0.020* |
| L4 | Streptomyces | 0.156 | 0.786 | 0.042* |
|  | Coprobacillus | 0.174 | 0.036* | 0.618 |
|  | Sphingomonas | 0.149 | 0.048* | 0.756 |
|  | Rhodococcus | 0.671 | 0.143 | 0.040* |
|  | Lactococcus | 0.001** | 0.001** | 0.244 |
|  | Dechloromonas | 0.041* | 0.074 | 0.800 |
|  | Zea | 0.065 | 0.998 | 0.042* |
|  | Proteus | 0.518 | 0.048* | 0.001** |
|  | Phenylobacterium | 0.041* | 0.083 | 0.683 |
|  | Anoxybacillus | 0.041* | 0.105 | 0.619 |
|  | Acetobacter | 0.065 | 0.001** | 0.332 |
| L21 | Ochrobactrum | 0.518 | 0.033* | 0.135 |
|  | Campylobacter | 0.055 | 0.998 | 0.031* |
|  | Proteus | 0.293 | 0.411 | 0.036* |
|  | Akkermansia | 0.001** | 0.062 | 0.618 |
|  | Dehalobacterium | 0.601 | 0.027* | 0.084 |
|  | Barnesiella | 0.097 | 0.033* | 0.712 |
|  | Amycolatopsis | 0.068 | 0.033* | 0.886 |
|  | Sphingobacterium | 0.065 | 0.033* | 0.904 |
| L42 | Bacteroides | 0.001** | 0.872 | 0.001** |
|  | Faecalibacterium | 0.065 | 0.497 | 0.012* |
|  | Campylobacter | 0.045* | 0.827 | 0.012* |
|  | Oscillospira | 0.068 | 0.786 | 0.015* |
|  | Ruminococcus | 0.079 | 0.827 | 0.031* |
|  | Zoogloea | 0.045* | 0.931 | 0.075 |
|  | AF12 | 0.045* | 0.001** | 0.590 |
|  | Megamonas | 0.126 | 0.036* | 0.756 |
|  | Coprococcus | 0.077 | 0.807 | 0.026* |
|  | Dorea | 0.112 | 0.762 | 0.031* |
|  | Coprobacillus | 0.133 | 0.616 | 0.031* |
|  | Butyricicoccus | 0.065 | 0.616 | 0.012* |
|  | cc_115 | 0.097 | 0.417 | 0.012* |
|  | Veillonella | 0.510 | 0.083 | 0.015* |
|  | Bilophila | 0.027* | 0.998 | 0.012* |
|  | Odoribacter | 0.041* | 0.795 | 0.012* |
|  | Rothia | 0.045* | 0.869 | 0.015* |
|  | Dehalobacterium | 0.112 | 0.786 | 0.035* |
|  | Clostridium | 0.068 | 0.586 | 0.012* |
|  | Marinobacter | 0.237 | 0.511 | 0.040* |
|  | Facklamia | 0.096 | 0.881 | 0.042* |
|  | Natronincola_Anaerovirgula | 0.078 | 0.884 | 0.036* |
|  | Alistipes | 0.055 | 0.855 | 0.015* |

**TABLE S6** The profile of experimental samples.

|  | | Breeds | | |
| --- | --- | --- | --- | --- |
| Stage | | Beijing Fatty (Individual numbers) | Shiqiza (Individual numbers) | Xianju (Individual numbers) |
| Maternal Hen(faeces) | | 4 | 4 | 4 |
| Embryo | 4 days (whole embryo) | 13 | 5 | 9 |
|  | 19 days (intestines) | 12 | 5 | 7 |
| Chick | 4 days (faeces) | 15 | 12 | 12 |
|  | 21days (faeces) | 15 | 12 | 11 |
|  | 42 days (faeces) | 15 | 10 | 11 |
| TOTAL | | 176 | | |

**TABLE S7** Summary of Sequencing data for all samples.

| **Samples** | **Effective reads count** | **High-quality reads count** | **Percentage** |
| --- | --- | --- | --- |
| **1** | 72,142 | 71,088 | 98.54% |
| **2** | 60,606 | 60,201 | 99.33% |
| **3** | 59,999 | 59,166 | 98.61% |
| **6** | 15,482 | 14,657 | 94.67% |
| **12** | 72,115 | 71,498 | 99.14% |
| **14** | 60,641 | 59,646 | 98.36% |
| **15** | 60,209 | 59,614 | 99.01% |
| **16** | 66,349 | 65,652 | 98.95% |
| **18** | 79,958 | 79,311 | 99.19% |
| **19** | 127,712 | 126,005 | 98.66% |
| **20** | 77,592 | 76,840 | 99.03% |
| **21** | 43,681 | 43,366 | 99.28% |
| **22** | 48,281 | 47,970 | 99.36% |
| **23** | 41,369 | 40,995 | 99.10% |
| **24** | 89,291 | 87,461 | 97.95% |
| **25** | 102,181 | 90,775 | 88.84% |
| **26** | 42,692 | 42,130 | 98.68% |
| **27** | 68,233 | 67,310 | 98.65% |
| **28** | 42,575 | 42,376 | 99.53% |
| **29** | 65,968 | 65,646 | 99.51% |
| **31** | 56,724 | 56,122 | 98.94% |
| **32** | 41,071 | 40,394 | 98.35% |
| **33** | 56,145 | 55,685 | 99.18% |
| **34** | 38,176 | 37,872 | 99.20% |
| **37** | 31,911 | 31,581 | 98.97% |
| **38** | 31,410 | 31,030 | 98.79% |
| **39** | 69,854 | 69,114 | 98.94% |
| **118** | 4,184 | 3,758 | 89.82% |
| **122** | 49,478 | 48,882 | 98.80% |
| **123** | 45,005 | 44,492 | 98.86% |
| **124** | 76,351 | 74,833 | 98.01% |
| **125** | 9,957 | 9,793 | 98.35% |
| **126** | 122,966 | 117,806 | 95.80% |
| **127** | 3,670 | 3,579 | 97.52% |
| **130** | 8,814 | 8,361 | 94.86% |
| **131** | 5,165 | 4,374 | 84.69% |
| **132** | 27,427 | 26,514 | 96.67% |
| **133** | 72,353 | 71,866 | 99.33% |
| **134** | 31,878 | 30,889 | 96.90% |
| **135** | 13,258 | 13,101 | 98.82% |
| **136** | 6,648 | 6,378 | 95.94% |
| **137** | 19,318 | 18,844 | 97.55% |
| **139** | 50,842 | 50,662 | 99.65% |
| **140** | 65,251 | 65,106 | 99.78% |
| **141** | 67,682 | 67,466 | 99.68% |
| **144** | 10,971 | 10,862 | 99.01% |
| **147** | 79,643 | 79,183 | 99.42% |
| **149** | 96,546 | 94,305 | 97.68% |
| **150** | 66,797 | 65,019 | 97.34% |
| **154** | 50,523 | 49,181 | 97.34% |
| **155** | 4,264 | 4,187 | 98.19% |
| **157** | 106,622 | 105,166 | 98.63% |
| **158** | 133,512 | 132,060 | 98.91% |
| **159** | 100,346 | 96,752 | 96.42% |
| **160** | 61,738 | 61,102 | 98.97% |
| **161** | 71,018 | 70,231 | 98.89% |
| **162** | 68,117 | 67,841 | 99.59% |
| **163** | 70,842 | 69,728 | 98.43% |
| **164** | 83,396 | 82,005 | 98.33% |
| **165** | 60,545 | 60,248 | 99.51% |
| **166** | 66,150 | 65,953 | 99.70% |
| **167** | 105,724 | 104,184 | 98.54% |
| **168** | 71,080 | 70,728 | 99.50% |
| **169** | 100,991 | 100,187 | 99.20% |
| **170** | 69,397 | 68,571 | 98.81% |
| **171** | 46,944 | 46,585 | 99.24% |
| **172** | 61,549 | 59,644 | 96.90% |
| **173** | 60,659 | 59,884 | 98.72% |
| **174** | 55,568 | 54,899 | 98.80% |
| **175** | 104,011 | 102,313 | 98.37% |
| **176** | 92,101 | 91,427 | 99.27% |
| **177** | 73,892 | 73,238 | 99.11% |
| **178** | 85,322 | 84,332 | 98.84% |
| **179** | 61,499 | 61,001 | 99.19% |
| **180** | 66,413 | 65,539 | 98.68% |
| **181** | 43,869 | 42,992 | 98.00% |
| **182** | 89,034 | 86,757 | 97.44% |
| **183** | 124,836 | 115,484 | 92.51% |
| **184** | 104,031 | 102,871 | 98.88% |
| **185** | 35,799 | 35,393 | 98.87% |
| **186** | 95,956 | 95,174 | 99.19% |
| **187** | 82,641 | 80,110 | 96.94% |
| **188** | 70,358 | 69,738 | 99.12% |
| **189** | 88,208 | 87,239 | 98.90% |
| **190** | 46,322 | 45,252 | 97.69% |
| **191** | 88,877 | 87,659 | 98.63% |
| **192** | 145,554 | 144,162 | 99.04% |
| **193** | 70,382 | 69,854 | 99.25% |
| **194** | 48,566 | 48,001 | 98.84% |
| **195** | 99,050 | 98,191 | 99.13% |
| **197** | 30,555 | 28,755 | 94.11% |
| **198** | 32,010 | 31,455 | 98.27% |
| **199** | 32,167 | 31,779 | 98.79% |
| **200** | 47,167 | 46,540 | 98.67% |
| **201** | 46,242 | 45,929 | 99.32% |
| **202** | 51,014 | 50,358 | 98.71% |
| **203** | 47,566 | 47,175 | 99.18% |
| **204** | 48,258 | 47,937 | 99.33% |
| **205** | 66,303 | 53,681 | 80.96% |
| **206** | 71,066 | 56,032 | 78.85% |
| **207** | 35,495 | 34,928 | 98.40% |
| **208** | 33,122 | 31,050 | 93.74% |
| **209** | 43,817 | 43,506 | 99.29% |
| **210** | 69,969 | 69,368 | 99.14% |
| **211** | 48,859 | 48,279 | 98.81% |
| **212** | 35,102 | 34,556 | 98.44% |
| **213** | 31,728 | 31,391 | 98.94% |
| **214** | 56,839 | 56,416 | 99.26% |
| **215** | 36,841 | 35,184 | 95.50% |
| **216** | 37,037 | 35,780 | 96.61% |
| **217** | 62,788 | 62,332 | 99.27% |
| **218** | 32,763 | 31,383 | 95.79% |
| **219** | 48,275 | 45,559 | 94.37% |
| **220** | 46,039 | 44,019 | 95.61% |
| **221** | 49,116 | 47,128 | 95.95% |
| **222** | 46,749 | 46,469 | 99.40% |
| **223** | 33,080 | 28,071 | 84.86% |
| **224** | 35,024 | 34,770 | 99.27% |
| **225** | 35,688 | 35,070 | 98.27% |
| **226** | 35,271 | 33,845 | 95.96% |
| **227** | 43,323 | 41,381 | 95.52% |
| **228** | 52,957 | 51,587 | 97.41% |
| **229** | 39,273 | 38,981 | 99.26% |
| **230** | 37,338 | 37,135 | 99.46% |
| **231** | 27,249 | 27,050 | 99.27% |
| **232** | 27,729 | 27,108 | 97.76% |
| **233** | 48,666 | 43,111 | 88.59% |
| **234** | 38,586 | 35,579 | 92.21% |
| **235** | 34,990 | 33,407 | 95.48% |
| **236** | 31,631 | 30,910 | 97.72% |
| **237** | 35,866 | 33,619 | 93.74% |
| **238** | 44,582 | 41,967 | 94.13% |
| **239** | 33,233 | 31,266 | 94.08% |
| **240** | 58,486 | 57,413 | 98.17% |
| **241** | 35,120 | 34,663 | 98.70% |
| **242** | 35,246 | 35,001 | 99.30% |
| **244** | 65,793 | 65,302 | 99.25% |
| **245** | 52,237 | 51,732 | 99.03% |
| **246** | 282,963 | 244,104 | 86.27% |
| **247** | 106,570 | 102,208 | 95.91% |
| **248** | 35,054 | 34,692 | 98.97% |
| **249** | 31,493 | 30,427 | 96.62% |
| **250** | 22,839 | 22,678 | 99.30% |
| **252** | 45,030 | 44,202 | 98.16% |
| **253** | 45,535 | 44,784 | 98.35% |
| **254** | 88,592 | 88,106 | 99.45% |
| **256** | 35,050 | 34,853 | 99.44% |
| **257** | 39,076 | 38,706 | 99.05% |
| **258** | 58,448 | 43,376 | 74.21% |
| **259** | 40,794 | 39,268 | 96.26% |
| **260** | 35,799 | 34,668 | 96.84% |
| **261** | 33,648 | 32,295 | 95.98% |
| **262** | 47,561 | 45,918 | 96.55% |
| **263** | 62,285 | 61,426 | 98.62% |
| **264** | 63,569 | 62,751 | 98.71% |
| **265** | 62,228 | 61,561 | 98.93% |
| **266** | 28,316 | 27,208 | 96.09% |
| **267** | 43,203 | 35,391 | 81.92% |
| **268** | 47,608 | 46,677 | 98.04% |
| **269** | 50,679 | 48,041 | 94.79% |
| **270** | 28,529 | 28,275 | 99.11% |
| **271** | 32,200 | 30,971 | 96.18% |
| **272** | 28,747 | 27,810 | 96.74% |
| **273** | 35,664 | 34,686 | 97.26% |
| **391** | 49,753 | 49,451 | 99.39% |
| **392** | 34,602 | 34,245 | 98.97% |
| **393** | 62,055 | 61,778 | 99.55% |
| **394** | 125,717 | 120,741 | 96.04% |
| **395** | 55,129 | 54,810 | 99.42% |
| **396** | 67,039 | 66,125 | 98.64% |
| **397** | 109,843 | 109,243 | 99.45% |
| **398** | 53,301 | 51,290 | 96.23% |
| **399** | 124,861 | 115,889 | 92.81% |
| **400** | 187,440 | 178,708 | 95.34% |
| **402** | 59,505 | 57,339 | 96.36% |
| **403** | 31,652 | 31,421 | 99.27% |
| **Total** | 10,182,909 | 9,887,520 | 97.10% |

**TABLE S8** Characteristics of microbial operation taxonomic units’ annotation results for all samples.

| **Sample** | **Phylum** | **Class** | **Order** | **Family** | **Genus** | **Species** |
| --- | --- | --- | --- | --- | --- | --- |
| **1** | 184 | 184 | 180 | 165 | 106 | 27 |
| **2** | 205 | 205 | 197 | 178 | 118 | 26 |
| **3** | 234 | 234 | 224 | 198 | 131 | 27 |
| **6** | 398 | 398 | 383 | 335 | 202 | 43 |
| **12** | 253 | 252 | 244 | 223 | 134 | 24 |
| **14** | 269 | 269 | 261 | 240 | 156 | 33 |
| **15** | 225 | 225 | 219 | 195 | 123 | 24 |
| **16** | 223 | 223 | 218 | 201 | 126 | 29 |
| **18** | 278 | 278 | 272 | 243 | 149 | 30 |
| **19** | 391 | 391 | 381 | 325 | 205 | 33 |
| **20** | 362 | 362 | 353 | 316 | 193 | 36 |
| **21** | 207 | 207 | 199 | 184 | 124 | 23 |
| **22** | 204 | 204 | 196 | 175 | 123 | 26 |
| **23** | 190 | 190 | 185 | 170 | 106 | 17 |
| **24** | 212 | 211 | 202 | 174 | 104 | 25 |
| **25** | 717 | 716 | 697 | 583 | 338 | 73 |
| **26** | 250 | 250 | 239 | 207 | 127 | 22 |
| **27** | 280 | 280 | 272 | 242 | 143 | 25 |
| **28** | 149 | 149 | 147 | 136 | 88 | 21 |
| **29** | 237 | 237 | 231 | 199 | 125 | 25 |
| **31** | 208 | 208 | 206 | 186 | 119 | 23 |
| **32** | 189 | 189 | 181 | 162 | 103 | 17 |
| **33** | 175 | 175 | 172 | 151 | 92 | 16 |
| **34** | 237 | 237 | 231 | 208 | 127 | 31 |
| **37** | 149 | 149 | 145 | 133 | 93 | 19 |
| **38** | 162 | 162 | 159 | 142 | 89 | 20 |
| **39** | 211 | 211 | 206 | 193 | 125 | 26 |
| **118** | 114 | 114 | 113 | 103 | 57 | 13 |
| **122** | 357 | 357 | 342 | 303 | 191 | 47 |
| **123** | 296 | 296 | 279 | 255 | 152 | 35 |
| **124** | 423 | 423 | 409 | 362 | 224 | 50 |
| **125** | 231 | 231 | 225 | 205 | 128 | 33 |
| **126** | 749 | 748 | 725 | 611 | 350 | 70 |
| **127** | 239 | 239 | 233 | 211 | 133 | 27 |
| **130** | 209 | 208 | 202 | 183 | 113 | 30 |
| **131** | 256 | 256 | 248 | 226 | 131 | 25 |
| **132** | 294 | 294 | 280 | 244 | 136 | 28 |
| **133** | 348 | 348 | 329 | 289 | 182 | 45 |
| **134** | 532 | 532 | 522 | 448 | 258 | 50 |
| **135** | 316 | 316 | 309 | 270 | 174 | 35 |
| **136** | 232 | 232 | 226 | 195 | 116 | 28 |
| **137** | 429 | 429 | 421 | 372 | 221 | 44 |
| **139** | 294 | 294 | 286 | 264 | 166 | 34 |
| **140** | 231 | 231 | 222 | 204 | 132 | 27 |
| **141** | 287 | 287 | 277 | 250 | 152 | 32 |
| **144** | 318 | 318 | 311 | 287 | 187 | 40 |
| **147** | 388 | 387 | 366 | 325 | 197 | 50 |
| **149** | 590 | 590 | 574 | 502 | 302 | 64 |
| **150** | 309 | 309 | 298 | 261 | 163 | 36 |
| **154** | 562 | 562 | 550 | 476 | 296 | 63 |
| **155** | 217 | 215 | 210 | 180 | 118 | 27 |
| **157** | 201 | 201 | 201 | 184 | 144 | 31 |
| **158** | 281 | 281 | 280 | 233 | 157 | 30 |
| **159** | 145 | 145 | 145 | 127 | 91 | 12 |
| **160** | 197 | 197 | 197 | 175 | 130 | 23 |
| **161** | 181 | 181 | 178 | 160 | 109 | 17 |
| **162** | 221 | 220 | 218 | 192 | 141 | 34 |
| **163** | 407 | 406 | 397 | 320 | 202 | 38 |
| **164** | 249 | 249 | 248 | 217 | 141 | 21 |
| **165** | 314 | 313 | 310 | 257 | 180 | 31 |
| **166** | 121 | 121 | 121 | 112 | 95 | 17 |
| **167** | 280 | 280 | 278 | 241 | 177 | 35 |
| **168** | 204 | 204 | 203 | 178 | 121 | 22 |
| **169** | 216 | 216 | 209 | 181 | 131 | 28 |
| **170** | 183 | 183 | 183 | 165 | 123 | 29 |
| **171** | 128 | 128 | 128 | 118 | 90 | 24 |
| **172** | 175 | 174 | 171 | 150 | 117 | 23 |
| **173** | 207 | 207 | 205 | 186 | 141 | 33 |
| **174** | 161 | 161 | 160 | 141 | 107 | 26 |
| **175** | 171 | 171 | 169 | 149 | 121 | 25 |
| **176** | 311 | 311 | 303 | 264 | 183 | 38 |
| **177** | 222 | 222 | 222 | 203 | 150 | 28 |
| **178** | 250 | 250 | 248 | 221 | 156 | 25 |
| **179** | 243 | 243 | 240 | 214 | 146 | 26 |
| **180** | 128 | 128 | 127 | 115 | 91 | 20 |
| **181** | 442 | 442 | 436 | 345 | 232 | 47 |
| **182** | 261 | 261 | 256 | 232 | 163 | 33 |
| **183** | 139 | 139 | 139 | 115 | 77 | 14 |
| **184** | 177 | 177 | 177 | 162 | 119 | 21 |
| **185** | 330 | 330 | 329 | 283 | 201 | 43 |
| **186** | 258 | 258 | 257 | 230 | 152 | 32 |
| **187** | 219 | 219 | 217 | 194 | 149 | 27 |
| **188** | 126 | 126 | 126 | 114 | 93 | 19 |
| **189** | 248 | 248 | 245 | 216 | 148 | 35 |
| **190** | 207 | 207 | 207 | 192 | 139 | 36 |
| **191** | 360 | 360 | 357 | 323 | 227 | 51 |
| **192** | 375 | 375 | 370 | 331 | 232 | 51 |
| **193** | 205 | 204 | 204 | 178 | 129 | 26 |
| **194** | 182 | 181 | 179 | 158 | 126 | 31 |
| **195** | 314 | 314 | 311 | 280 | 203 | 37 |
| **197** | 647 | 645 | 642 | 494 | 304 | 58 |
| **198** | 561 | 560 | 558 | 408 | 228 | 37 |
| **199** | 636 | 635 | 633 | 471 | 271 | 50 |
| **200** | 514 | 514 | 513 | 379 | 215 | 39 |
| **201** | 365 | 365 | 364 | 290 | 186 | 50 |
| **202** | 629 | 629 | 628 | 459 | 261 | 43 |
| **203** | 521 | 520 | 520 | 384 | 227 | 39 |
| **204** | 343 | 343 | 342 | 263 | 164 | 36 |
| **205** | 395 | 395 | 395 | 282 | 139 | 19 |
| **206** | 389 | 389 | 389 | 277 | 139 | 21 |
| **207** | 304 | 303 | 303 | 239 | 154 | 36 |
| **208** | 174 | 174 | 174 | 145 | 107 | 15 |
| **209** | 392 | 392 | 392 | 305 | 177 | 36 |
| **210** | 468 | 468 | 468 | 351 | 211 | 44 |
| **211** | 234 | 234 | 232 | 184 | 126 | 32 |
| **212** | 184 | 184 | 183 | 138 | 105 | 33 |
| **213** | 404 | 404 | 404 | 304 | 186 | 32 |
| **214** | 479 | 479 | 477 | 367 | 231 | 43 |
| **215** | 248 | 248 | 248 | 201 | 148 | 27 |
| **216** | 445 | 445 | 445 | 348 | 226 | 53 |
| **217** | 665 | 664 | 663 | 504 | 285 | 48 |
| **218** | 580 | 580 | 579 | 432 | 258 | 39 |
| **219** | 201 | 201 | 201 | 160 | 117 | 10 |
| **220** | 372 | 372 | 372 | 279 | 172 | 33 |
| **221** | 438 | 438 | 436 | 331 | 191 | 40 |
| **222** | 429 | 429 | 427 | 324 | 214 | 44 |
| **223** | 355 | 355 | 352 | 275 | 185 | 39 |
| **224** | 479 | 479 | 476 | 374 | 234 | 44 |
| **225** | 481 | 480 | 477 | 377 | 239 | 49 |
| **226** | 712 | 711 | 696 | 569 | 345 | 73 |
| **227** | 417 | 416 | 413 | 341 | 201 | 49 |
| **228** | 358 | 358 | 350 | 293 | 183 | 46 |
| **229** | 421 | 420 | 415 | 318 | 177 | 39 |
| **230** | 471 | 471 | 470 | 375 | 230 | 52 |
| **231** | 396 | 396 | 395 | 278 | 159 | 35 |
| **232** | 298 | 298 | 296 | 234 | 142 | 32 |
| **233** | 440 | 440 | 439 | 314 | 171 | 28 |
| **234** | 365 | 365 | 364 | 271 | 164 | 34 |
| **235** | 276 | 276 | 274 | 241 | 173 | 33 |
| **236** | 334 | 334 | 332 | 274 | 175 | 37 |
| **237** | 229 | 229 | 228 | 192 | 137 | 27 |
| **238** | 319 | 319 | 318 | 256 | 166 | 46 |
| **239** | 354 | 353 | 350 | 296 | 194 | 39 |
| **240** | 480 | 480 | 479 | 383 | 235 | 49 |
| **241** | 204 | 204 | 203 | 161 | 115 | 36 |
| **242** | 275 | 275 | 274 | 213 | 127 | 29 |
| **244** | 296 | 296 | 295 | 262 | 187 | 44 |
| **245** | 321 | 321 | 321 | 264 | 173 | 43 |
| **246** | 215 | 214 | 214 | 193 | 150 | 34 |
| **247** | 514 | 513 | 511 | 374 | 205 | 38 |
| **248** | 276 | 275 | 270 | 234 | 167 | 40 |
| **249** | 458 | 457 | 455 | 366 | 234 | 51 |
| **250** | 448 | 448 | 447 | 340 | 184 | 32 |
| **252** | 517 | 517 | 517 | 377 | 196 | 40 |
| **253** | 548 | 547 | 543 | 404 | 222 | 37 |
| **254** | 541 | 541 | 539 | 397 | 213 | 37 |
| **256** | 363 | 363 | 363 | 279 | 151 | 28 |
| **257** | 336 | 336 | 333 | 249 | 139 | 32 |
| **258** | 463 | 463 | 463 | 325 | 165 | 22 |
| **259** | 222 | 222 | 221 | 185 | 136 | 32 |
| **260** | 390 | 390 | 387 | 302 | 197 | 48 |
| **261** | 175 | 175 | 172 | 148 | 102 | 28 |
| **262** | 235 | 235 | 232 | 193 | 145 | 31 |
| **263** | 543 | 543 | 539 | 397 | 221 | 40 |
| **264** | 641 | 641 | 632 | 498 | 282 | 51 |
| **265** | 429 | 429 | 421 | 375 | 243 | 52 |
| **266** | 231 | 231 | 228 | 193 | 136 | 33 |
| **267** | 421 | 421 | 421 | 300 | 146 | 25 |
| **268** | 222 | 222 | 222 | 179 | 133 | 34 |
| **269** | 177 | 177 | 176 | 152 | 119 | 30 |
| **270** | 246 | 246 | 245 | 187 | 124 | 30 |
| **271** | 214 | 214 | 214 | 174 | 126 | 32 |
| **272** | 465 | 465 | 465 | 338 | 181 | 33 |
| **273** | 281 | 281 | 281 | 213 | 146 | 36 |
| **391** | 243 | 243 | 243 | 219 | 159 | 28 |
| **392** | 601 | 601 | 600 | 473 | 316 | 53 |
| **393** | 627 | 626 | 621 | 488 | 295 | 51 |
| **394** | 535 | 534 | 532 | 444 | 308 | 44 |
| **395** | 551 | 550 | 541 | 455 | 297 | 55 |
| **396** | 462 | 461 | 460 | 385 | 276 | 38 |
| **397** | 717 | 716 | 715 | 570 | 338 | 57 |
| **398** | 444 | 443 | 443 | 353 | 234 | 29 |
| **399** | 772 | 771 | 766 | 603 | 358 | 51 |
| **400** | 906 | 905 | 899 | 720 | 426 | 69 |
| **402** | 296 | 296 | 294 | 261 | 206 | 25 |
| **403** | 496 | 495 | 495 | 391 | 247 | 28 |
