# Supplementary material for: Inheritance and Establishment of Gut Microbiota in Chickens
Source: Front Microbiol. 2017 Oct 10;8:1967. doi: 10.3389/fmicb.2017.01967 (PMC5641346; doi:10.3389/fmicb.2017.01967)
Supplement: Supplementary file 2 [file Image_1.PDF]

Supplemental Figures for "Inheritance and establishment of gut microbiota in chickens"

Supplemental Figures

Figure S1-S8

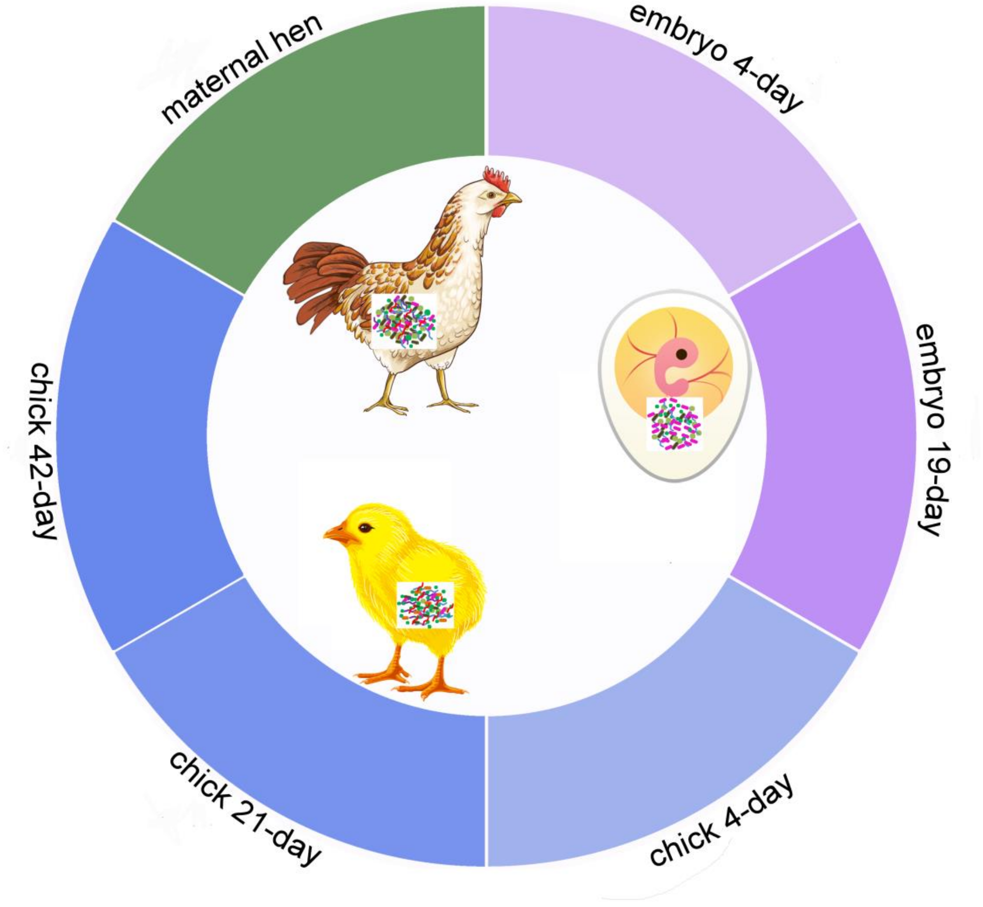

Figure S1 The individuals used in these experiments (please refer to supplemental material Table S6 for details). Ring chart reflects different stages. Every stage includes three native Chinese breeds: Beijing Fatty (B), Shiqiza (C) and Xianju (X) chickens.

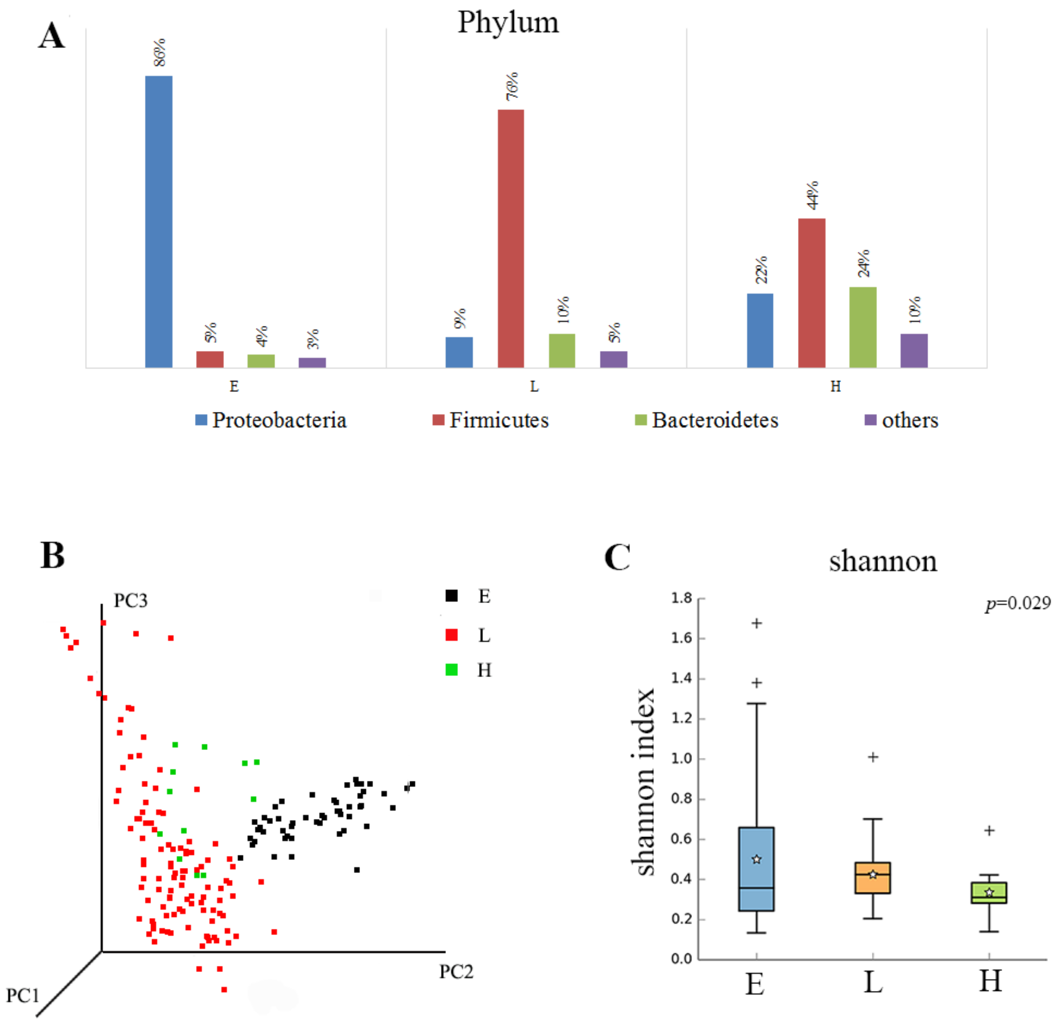

Figure S2 Comparison of microbiota detected from chick embryo (E), chick (L), and maternal hen (H). (**A**) Aggregate microbiota composition at phylum among different stages. (**B**) PCA plot display the distribution of microbiota: embryo is black, chick is red, and maternal hen is green. (**C**) Microbial diversity in embryo, chick, and hen.

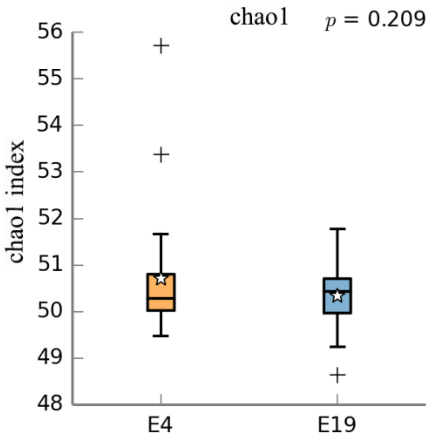

Figure S3 Microbial alpha diversity with a Box plot exhibiting the community richness (The Chao1 estimator).

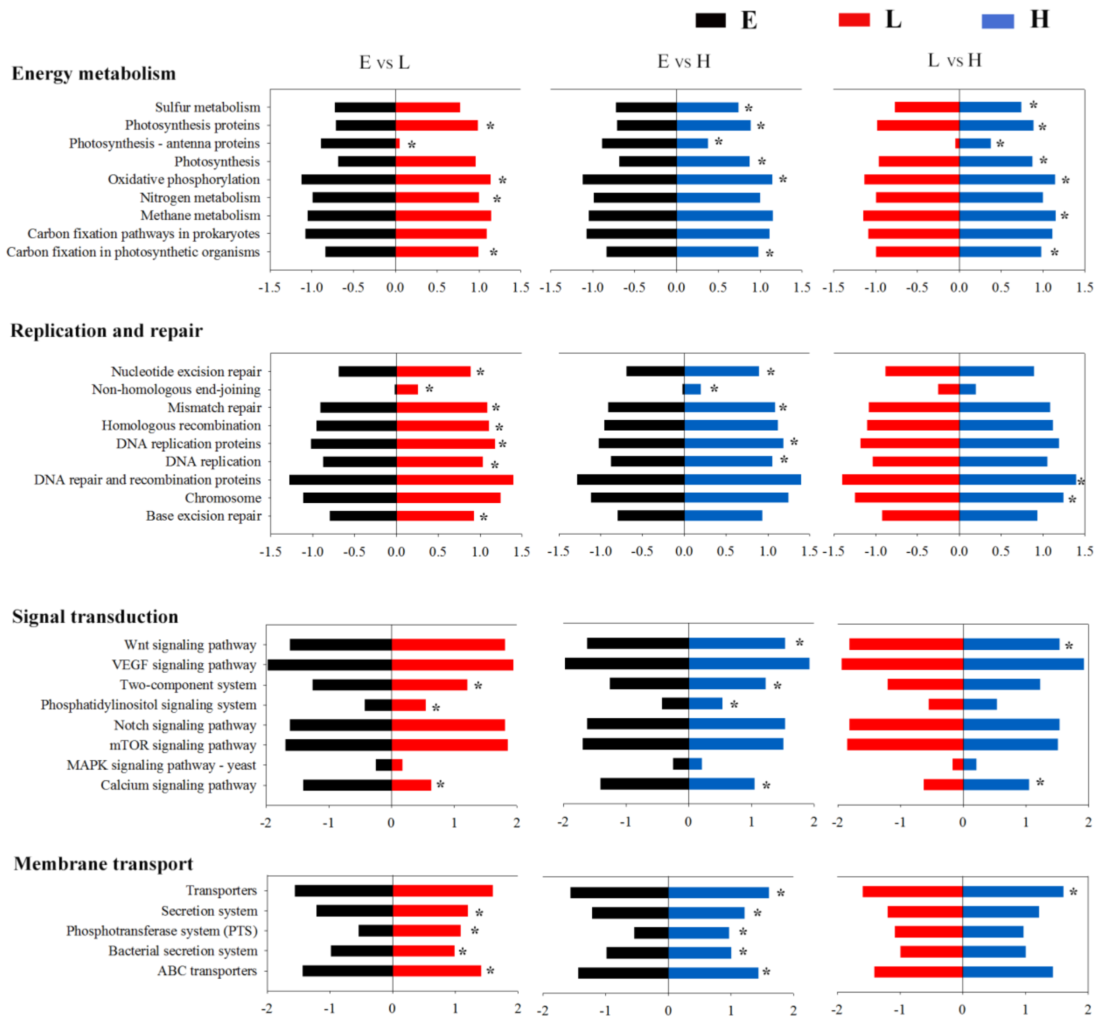

Figure S4 Significant microbial metabolism pathways in embryos (E), chicks (L) and maternal hens (H). Only major modules of KEGG pathways were shown (*p<0.05).

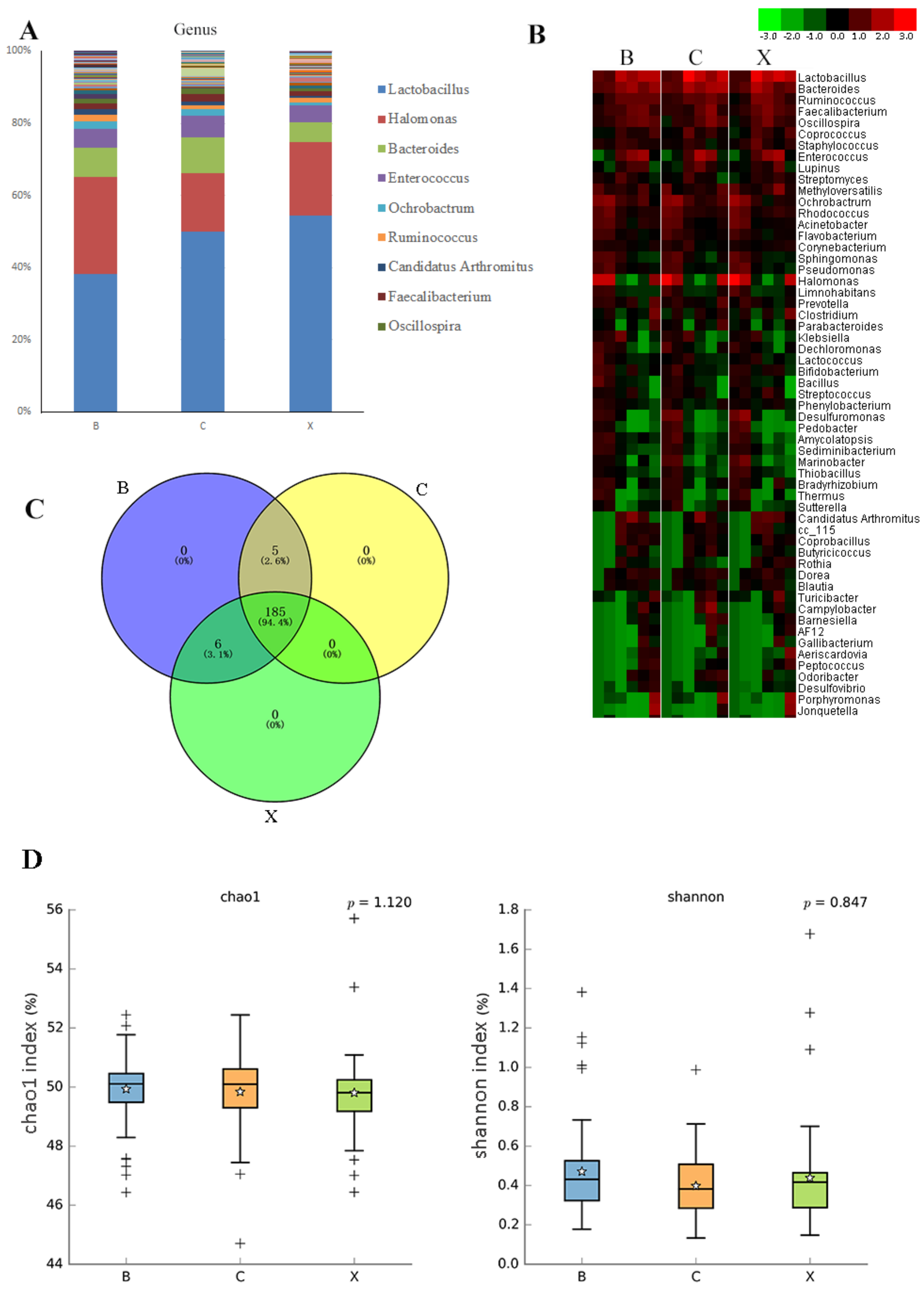

Figure S5 Comparison of genus microbiota obtained from Beijing Fatty (B), Xianju (X) and Shiqiza (C) chickens. (**A**) Aggregate microbiota composition at genus among three breeds. (**B**) Heatmap of hierarchy cluster results for the abundance of genus in

breeds. Colors reflect relative abundance from low (green) to high (red) (Color figure online). (**C**) The Venn diagram displayed the number of microbiota shared within different chicken breeds. (**D**) Microbial alpha diversity among Beijing Fatty, Xianju and Shiqiza chickens.

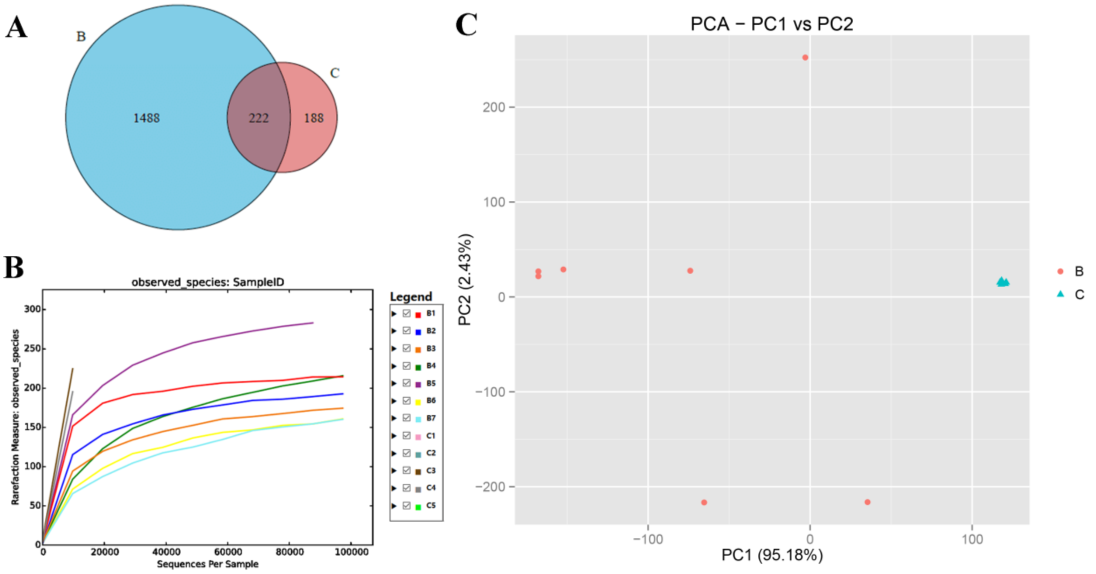

Figure S6. The results of preliminary experiments. (**A**) Rarefaction curve under 97% similarity in the experimental group (B) and control group (C). (**B**) The Venn diagram displayed the number of the microbial operation taxonomic units (OTUs) shared in two groups. (**C**) Principal component plot displayed the distance of microbial distribution between the two groups.

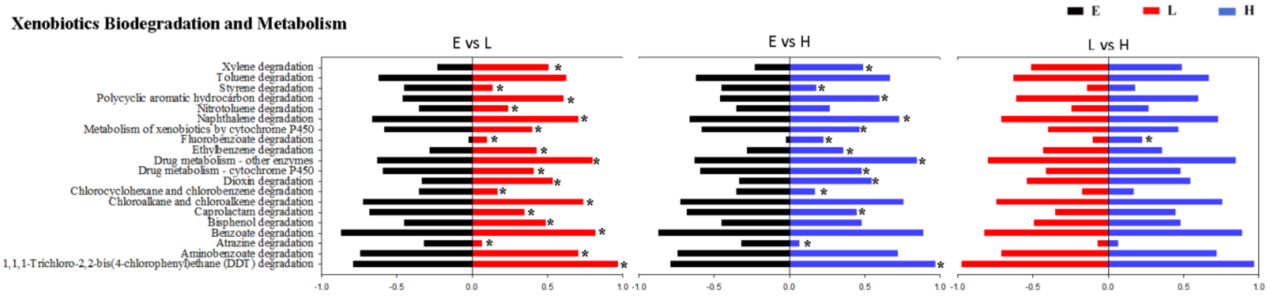

Figure S7 Significant microbial metabolism pathway of xenobiotics biodegradation and metabolism in embryos (E), chicks (L) and maternal hens (H) (*p<0.05).

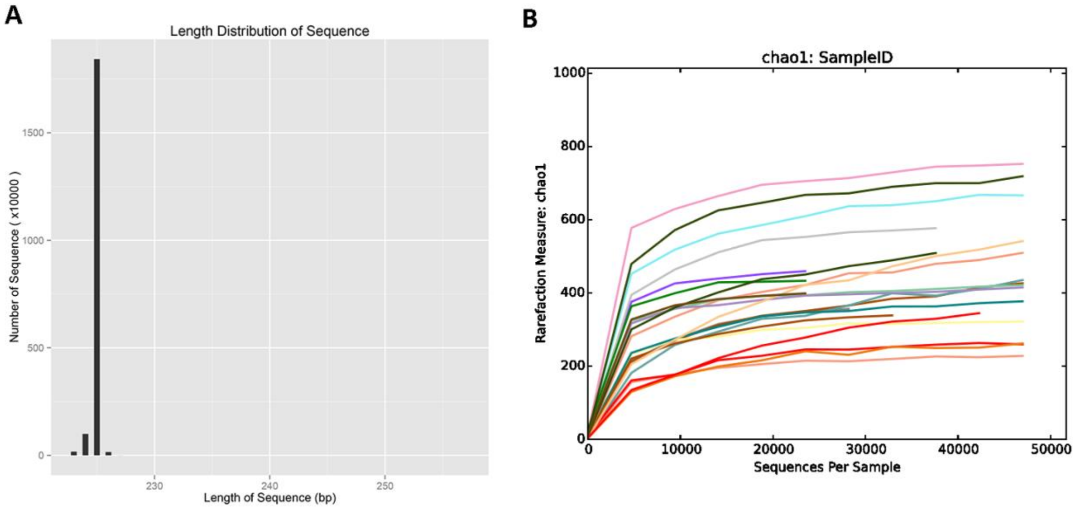

Figure S8. (A) The length distribution of high quality sequence. **(B)** Rarefaction curves under 97% similarity in all samples.
